# Dose-Response Modelling of Paralytic Shellfish Poisoning (PSP) in Humans

**DOI:** 10.3390/toxins10040141

**Published:** 2018-03-28

**Authors:** Nathalie Arnich, Anne Thébault

**Affiliations:** Risk Assessment Department, ANSES (French Agency for Food, Environmental and Occupational Health & Safety), 94701 Maisons-Alfort, France

**Keywords:** marine biotoxins, PSP, saxitoxin, STX, dose-response, ordinal modelling

## Abstract

Paralytic shellfish poisoning (PSP) is caused by a group of marine toxins with saxitoxin (STX) as the reference compound. Symptoms in humans after consumption of contaminated shellfish vary from slight neurological and gastrointestinal effects to fatal respiratory paralysis. A systematic review was conducted to identify reported cases of human poisoning associated with the ingestion of shellfish contaminated with paralytic shellfish toxins (PSTs). Raw data were collected from 143 exposed individuals (113 with symptoms, 30 without symptoms) from 13 studies. Exposure estimates were based on mouse bioassays except in one study. A significant relationship between exposure to PSTs and severity of symptoms was established by ordinal modelling. The critical minimal dose with a probability higher than 10% of showing symptoms is 0.37 µg STX eq./kg b.w. This means that 10% of the individuals exposed to this dose would have symptoms (without considering the severity of the symptoms). This dose is four-fold lower than the lowest-observed-adverse-effect-level (LOAEL) established by the European Food Safety Authority (EFSA, 2009) in the region of 1.5 μg STX eq./kg b.w. This work provides critical doses that could be used as point of departure to update the acute reference dose for STX. This is the first time a dose-symptoms model could be built for marine toxins using epidemiological data.

## 1. Introduction

Paralytic shellfish poisoning (PSP) is caused by a group of marine biotoxins with saxitoxin (STX) as the reference compound. To date, more than 50 compounds have been reported [1]. Paralytic shellfish toxins (PSTs) are mainly produced by marine dinoflagellates of the genus *Alexandrium*, e.g., *A. tamarense*, *A. minutum*, *A. catenella*, and by other dinoflagellates, such as *Pyrodinium bahamense* and *Gymnodinium catenatum*. PSTs are also produced by freshwater cyanobacteria of the genera *Anabaena*, *Cylindrospermopsis*, *Aphanizomenon*, *Planktothrix*, and *Lyngbia* [1,2]. PSTs can accumulate in filter-feeding bivalve molluscs such as oysters, mussels, scallops, and clams from various parts of the world. The toxins generally do not cause any adverse effects in the shellfish (in some cases filtration behavior is affected), and do not give any colour or taste that could alert the consumer. In addition, PSTs are heat-stable in shellfish at temperatures relevant for cooking [2].

In Europe, PSTs are regulated by the Commission Regulation (EC) No 853/2004, with a limit in live bivalve molluscs of 800 micrograms per kilogram, expressed as STX equivalents. This limit corresponds with most limits established in countries outside the EU, although these are often expressed differently as μg/100 g or mg/kg [2], such as Australia (Food Standards Australia New Zealand (FSANZ) Food Standard 1.4.1, [3]), Canada (Canadian standards (maximum levels) [4]), and the U.S. (chapter 6: natural toxins, [5]).

PSTs are neurotoxic. They bind to the voltage-gated sodium channels and block conduction of action potential, leading to a progressive loss of neuromuscular functions [1,2]. Symptoms of human intoxication vary from slight neurological and gastrointestinal effects (tingling sensation or numbness around the lips, headache, dizziness, nausea) to death by respiratory arrest [1,2]. In 2009, the European Food Safety Authority (EFSA) released a scientific opinion on the saxitoxin (STX)-group toxins [2]. From the available reports on intoxications in humans, comprising more than 500 individuals, a lowest-observed-adverse-effect-level (LOAEL) in the region of 1.5 μg STX equivalents/kg b.w. could be established. Since many individuals did not suffer adverse reactions at higher intakes it was expected that this LOAEL was close to the threshold for effects in sensitive individuals. However, this LOAEL was established following a qualitative approach.

In the present work, we developed a quantitative approach to (i) model the dose-response relationship between the amount of PSTs ingested and the severity of the symptoms in humans and (ii) identify a threshold for symptoms in individuals. To our knowledge, this is the first time that a quantitative model is developed for PSTs.

For that objective, data on intoxications in humans were identified through a systematic review and individual data were extracted. Exposure has been estimated based on a mouse bioassay analysis of contaminated shellfish (AOAC method 959.08) except for one study based on HPLC-FLD analysis. Data on exposure and symptoms were analysed with an ordinal model, and we used the model to predict the risk of different kind of symptoms associated with different doses, in a manner close to the estimation of a benchmark dose (BMD). This work provides critical doses that could be used as point of departure to update the acute reference dose (ARfD) for the PSTs. Currently, the ARfD established by EFSA is of 0.5 μg STX equivalents/kg b.w., a factor of three was considered sufficient to move from the LOAEL to an estimated no-observed-adverse-effect level (NOAEL) of 0.5 μg STX equivalents/kg b.w. No additional factor for variation among humans was deemed necessary because the data covered a large number of affected consumers, including sensitive individuals. This ARfD is consistent with the provisional value of 0.7 μg/kg b.w. set in 2004 by the Joint FAO/IOC/WHO ad hoc Expert Consultation on Biotoxins in Bivalve Molluscs [6], based on a LOAEL of 2 µg STX equivalent/kg b.w. and a safety factor of three because documentation of human cases includes a wide spectrum of people (occupation, age, and sex) and mild illness is readily reversible.

## 2. Results

### 2.1. Dose-Response Modelling

As a first step, we explored the relationship between dose and severity of symptoms with a logit or a probit model (Table 1). Each model is significant in comparison with the null model, with more than two units of difference. For both types of model (logit or probit), log10 transformation of the doses gives best fitting (lower AIC value).

**Figure 1:**
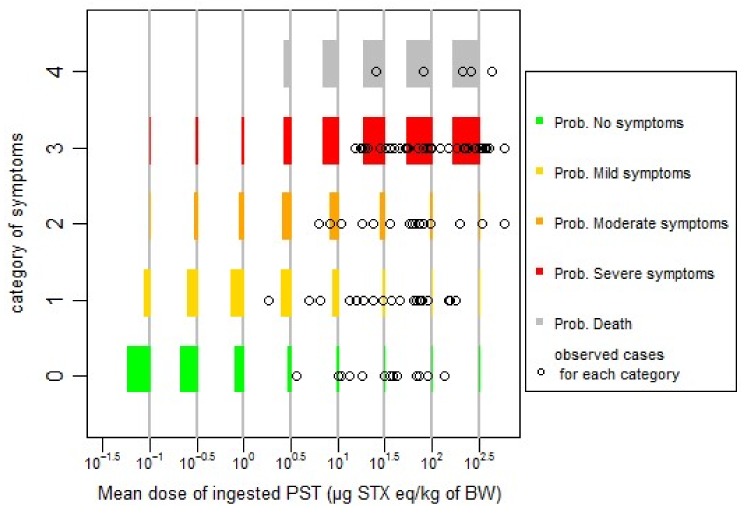
Predicted probabilities of specified symptoms for different level of doses with the CLMM model in comparison with the observed data.

**Figure 2:**
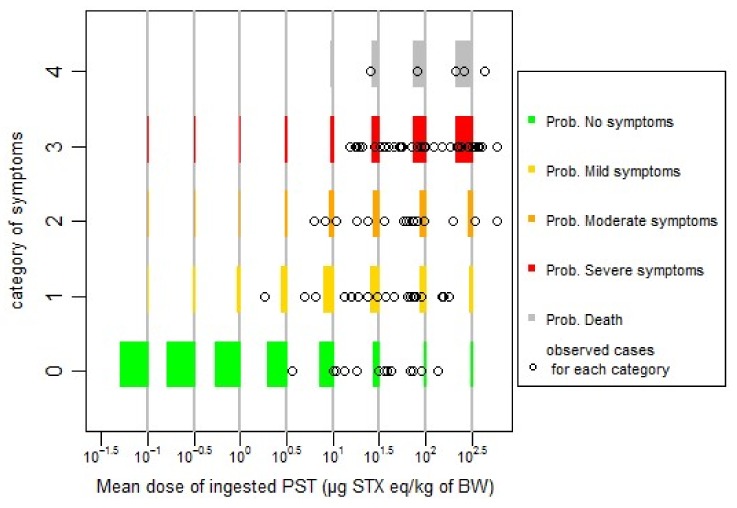
Predicted probabilities of specified symptoms for different level of doses with the CLM model in comparison with the observed data.

Based on the lowest AIC value, the difference between the two models (probit or logit) is not significant (AIC difference less than two units). For simplicity reasons, and based on the lowest value of AIC, we chose to fit our data with the probit model, and log10(dose), centred and reduced (standardized) for the next steps of the work. The effect of age or sex was not found to be significant at *p* = 0.05. The effect of publication as random effect (cumulative link mixed model, CLMM) gives the lowest AIC. However, the prediction of this last model under estimates no symptomatic cases, and overestimates symptomatic cases at low doses (Figure 1), in comparison with the model without random effect (Figure 2) (cumulative link model, CLM). There is a trade-off to make between the model complexity, the information given by the fitting and the available data, probably under-estimating no symptomatic cases at low dose. We chose, for simplicity reasons, to continue with the model without random effects (CLM model, Figure 2). 

The quality of fitting of the models can be checked graphically in Figure 2, for a cumulative link model without random effects (CLM model), and, in Figure 1, for the model with random effects (CLMM model).

From Figure 2, we can see a relatively good agreement between the predicted and observed data. At low dose, lower than 10 µg STX eq/kg b.w, probability of being asymptomatic or showing mild symptoms are the main probable situation in agreement with the observations.

The parameters of the final model (CLM model) are given in Table 2.

**Figure 3:**
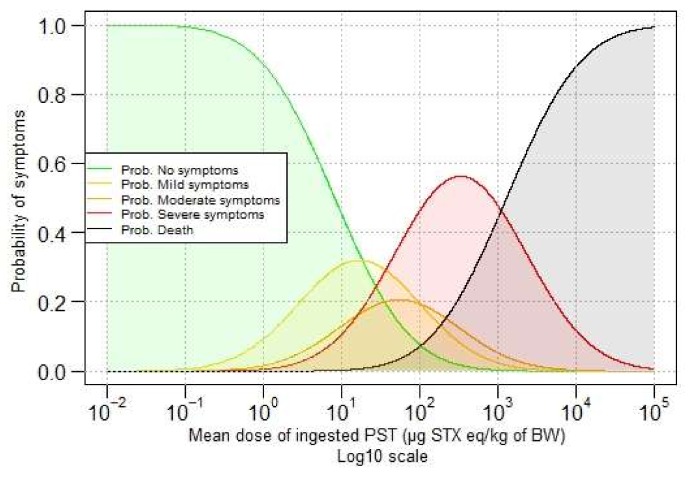
Predicted probabilities from the ordinal model of showing symptoms in function of the dose of ingested PSTs (log10 scale) with five categories of symptoms.

Predicted probabilities for different doses are given in Figure 3, which shows that the probability of not showing symptoms decreases when the ingested dose of PST increases. In contrast, the probability of death increases when the ingested dose of PST increases.

Table 3 gives the probability of showing symptoms (or not) in function of the dose of ingested PSTs with five categories of symptoms. For example, at the ingested dose of 1 µg STX eq/kg b.w., there is a probability of 88.7% of having no symptoms, 9.2% to develop mild symptoms, and 0.002% of death.

### 2.2. Critical Doses

**Figure 4:**
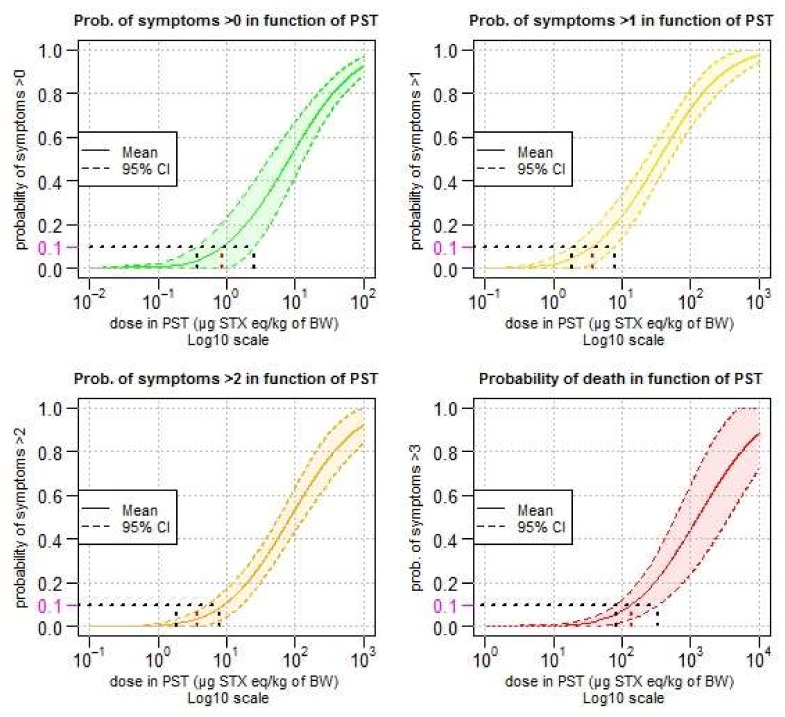
Predicted probability of symptoms more than a specified level in function of the mean dose of ingested PSTs (log10 scale). Lower critical dose (LCD) (for the lower bound of the confidence interval at 95%) (the black dotted segment, left of the dotted red segment), critical dose (CD) (mean) (the dotted red segment), and upper critical dose (UCD) (the upper bound of the confidence interval at 95%) (the black dotted segment right of the red segment), are represented for a risk of 10%. Category of symptoms: 0 = no symptoms; 1 = mild symptoms; 2 = moderate symptoms; 3 = severe symptoms; 4 = death. CI = Confidence Interval.

Confidence intervals of the predicted cumulative distribution for category of symptoms other than a specified level are presented in Figure 4.

The lower critical doses (LCD) (corresponding to the lower-bound of the confidence interval at 95%) is comparable to the BMDL approach (lower bound of the benchmark dose confidence interval). The lower critical dose with a probability higher than 10% of showing symptoms is 0.37 µg STX eq./kg b.w. (Figure 4, top left). This means that 10% of the individuals exposed to this dose of 0.37 µg STX eq./kg b.w. would have symptoms (without considering the severity of the symptoms). For mild symptoms, this value is 1.85 µg STX eq./kg b.w. (Figure 4, top right). For moderate symptoms (Figure 4, bottom left) and death (Figure 4, bottom right), the respective values are 5.16 and 82.2 µg STX eq./kg b.w. Estimated critical doses (including lower and upper critical doses) are listed in Table 4.

## 3. Discussion

The main objectives of our study were to develop a quantitative approach to (i) model the dose-response relationship between the amount of PSTs ingested and the severity of the symptoms in humans, and (ii) identify a threshold for symptoms in individuals.

Until now, only a qualitative approach has been used to establish a lowest-observed-adverse-effect-level (LOAEL). In 2009, the European Food Safety Authority (EFSA) identified a LOAEL in the region of 1.5 μg STX equivalents/kg b.w. from the available reports on intoxications in humans, comprising more than 500 individuals [2]. Since many individuals did not suffer adverse reactions at higher intakes it was expected that this LOAEL was close to the threshold for effects in sensitive individuals. In 2004, the Joint FAO/IOC/WHO ad hoc Expert Consultation on Biotoxins in Bivalve Molluscs [6] set a LOAEL of 2 µg STX equivalent/kg b.w. based on documentation of human cases including a wide spectrum of people (occupation, age and sex).

For our study, a systematic review was conducted to identify and collect raw data of reported cases of human poisoning associated with the ingestion of shellfish contaminated with PSTs. The aim of a systematic review is to minimize bias and maximize transparency to allow a more reliable review of the weight of evidence. This systematic review was conducted according to EFSA guidance [7]. Data from 30 studies and 329 exposed individuals were collected and extracted. In many cases, key information was missing (such as body weight, amount of contaminated shellfish consumed, and quantification of PSTs in shellfish) and, when possible, assumptions were made to include the case (for example, by using a default body of 60 kg for adults). In total, data for 143 exposed individuals (113 with symptoms, 30 without symptoms) from 13 studies were analysed.

According to our model (probit function), the minimal dose with the probability higher than 10% of showing symptoms is 0.37 µg STX eq./kg b.w. This means that 10% of the individuals exposed to this dose of 0.37 µg STX eq./kg b.w. would have symptoms (without considering the severity of the symptoms). This dose is four-fold lower than the lowest-observed-adverse-effect-level (LOAEL) established by EFSA in the region of 1.5 μg STX eq./kg b.w. [2].

The minimal dose with a probability higher than 10% of showing mild symptoms (tingling sensation or numbness around the lips gradually spreading to the face and neck, prickly sensation in fingertips and toes, headache, dizziness, and nausea) is 1.85 µg STX eq./kg b.w.

For moderate symptoms (incoherent speech, general weakness, slight respiratory difficulty, and rapid pulse) and fatal cases, the values are 5.16 and 82.2 µg STX eq./kg b.w., respectively.

To update and improve our modelling of the dose-response relationship for PSTs, we recommend future authors who will report human cases of PSP intoxication to include actual individual body weight, age, sex, reliable exposure estimate (amount of toxin ingested, number of shellfish, PST concentration in leftover meals). Data on exposure of individual who ate some shellfish but had no symptoms are also very important, in order to better model the dose-response relationship at low doses and get a more accurate estimate of the dose without symptoms. Even if low doses are included in our dataset (Figure 2) from different outbreaks, there is a publication bias on no symptomatic individuals, and it is possible that our dose-response could over-estimate the risk.

The mouse bioassay was the only method used for quantification of PSTs in shellfish in the outbreak data used in our model, except in one study [8] based on HPLC-FLD. However, the authors did not mention if they applied toxic equivalency factors to the sum of analogues found (GTX2 and 3) to express the concentration in µg STX equivalent/kg b.w. The use of MBA for quantification of PSTs in contaminated shellfish may result in an over- or under-estimation of the risk of showing symptoms in our dose-response modelling. We encourage the use of chemical methods (HPLC-FLD, AOAC method 2005.06) instead of the mouse bioassay (AOAC method 959.08), in order to be more informative on the toxin profile and more accurate when comparing results. The potency of PSTs in shellfish estimated by the mouse bioassay (administration by intraperitoneal injection) does not adequately represent the potency for human oral exposure. Using a single conversion factor of 0.18 to convert concentrations in mouse units to concentrations in µg STX equivalent/kg is a rough simplification, however, the uncertainty about this conversion factor is unknown, making any sensitivity analysis impossible. This conversion factor of 0.18 has been used by some authors of the selected studies [9,10] and by EFSA in its 2009 opinion on PSP, adopted by a panel of European experts [2].

In few of the selected outbreak studies (3/13), there was a chemical analysis of some samples of contaminated shellfish (and in human tissues), in order to identify the major analogues of PSTs [11,12,13], but this information was not used for contamination quantification of shellfish and cannot be used for dose-response modelling. Detailed information is provided in Table A1 in the Appendix. It is probably a limitation for inter-comparison between studies, but is not limitative for a first approach of the dose-response based on the overall toxicity. The identification of dinoflagellate was not systematically reported in outbreaks, but in Table A1 in the Appendix we reported the dinoflagellates associated with the outbreak, as indicated in the publication. Whenever possible, the case-outbreak report should provide all the available information to describe the situation of the outbreak, in its environmental context.

Data from quantification by HPLC-FLD, with or without corrective factor (toxic equivalency factor, TEF), should be tested in a similar dose-response modelling.

It could be of interest to have an iterative process and to include every new documented outbreak associated with PSTs in the future. Due to the small sample size of our dataset the model was estimated with all available data, and it was then not possible to make a cross-validation examination. With accurate new data, cross-validation or validation of the dose-response could be feasible [14].

It will also be interesting to make the prediction of cases given to the dose-response with real data of contamination, observed in contaminated areas, in order to evaluate if the prediction gives realistic values and if the management strategy (closure of a shellfish area) is efficient to avoid human cases of poisoning. 

This dose-response modelling is part of a wider research project on PST contamination in oysters (2014–2018). Modelling of contamination in oysters was developed to account for (i) the kinetics of accumulation and detoxification by oysters; (ii) the individual variability in diploid oyster contamination; and (iii) the temporal variability. The combination of two components (dose-response and oyster contamination) is in progress to perform a quantitative risk assessment for human consumers.

## 4. Materials and Methods

### 4.1. Systematic Review to Identify and Collect Raw Data of Reported Cases of Human Poisoning Associated with the Ingestion of Shellfish Contaminated with PSTs

#### 4.1.1. Principles of a Systematic Review

A systematic review aims to minimize bias and maximize transparency to allow a more reliable review of the weight of evidence. The whole process is adequately documented to allow others to critically appraise the judgments made in study selection and the collection, analysis, and interpretation of the results and, if necessary, to repeat or update the systematic review. According to the principles established by the Cochrane Institute (http://handbook.cochrane.org/) and EFSA (2010) [7], a systematic review is a rigorous scientific process and consists of several steps:

- search all existing studies published;
- assess the quality of each study and select those that meet a high standard of quality;
- synthesize the results of the selected studies; and
- if the data permit, perform statistical analysis (meta-analysis).

#### 4.1.2. Details of the Systematic Review Conducted in the Present Work

Evaluation of the effect of exposure on a population follows a structure called PECO:

P = population (human consumer of shellfish);
E = exposure (shellfish contaminated by PSP toxins);
C = comparator, the reference scenario; and
O = outcome (dose of PSP ingested, symptoms, % of ill persons).

Inclusion criteria were as follows:

- human outbreak;
- linked to consumption of shellfish;
- shellfish contaminated by PSP toxins;
- description of symptoms; and
- concentration of PSP toxins in shellfish and/or dose ingested.

Exclusion criteria were as follows:

- other toxins than PSP toxins;
- no data on concentration of PSP toxins in shellfish or dose ingested;
- no description of symptoms;
- other language than English or French; and
- review article.

The literature search was conducted on PUBMED and SCOPUS in March 2016 and updated in February 2017 and February 2018.

On PUBMED, the request was: ((paralytic shellfish poisoning) OR saxitoxins) AND (illness OR foodborne); 23 March 2016; 479 records were found.

On SCOPUS the request was: (TITLE-ABS-KEY (paralytic shellfish poisoning) AND TITLE-ABS-KEY (saxitoxin*) OR TITLE-ABS-KEY (illness) OR TITLE-ABS-KEY (disease*)); 23 March 2016; 635 records were found.

**Figure 5:**
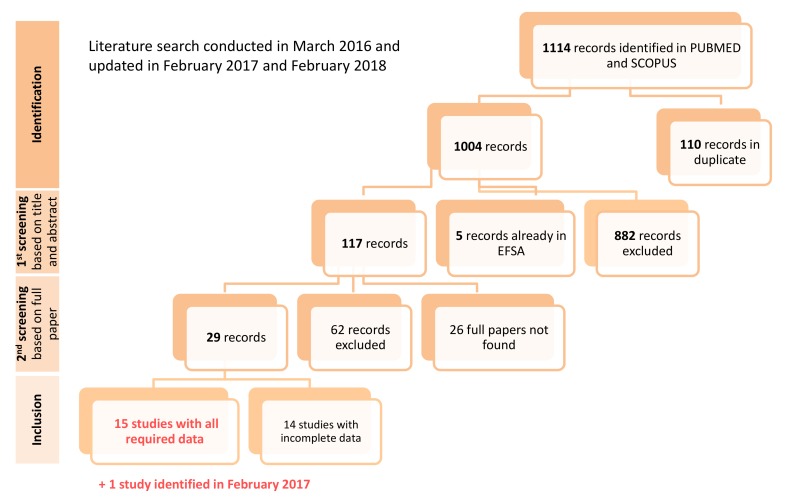
Diagram of the literature search of reported cases of human poisoning associated with the ingestion of shellfish contaminated with PSTs.

The diagram of the literature search of reported cases of human poisoning associated with the ingestion of shellfish contaminated with PSTs is given in Figure 5.

Individual data were collected from 30 studies for 329 exposed individuals. In many cases, key information was missing:

- total number of persons exposed;
- body weight of ill person;
- amount of shellfish ingested; and
- in some cases, contamination was estimated in shellfish collected several days or weeks after the outbreak.

When possible, assumptions were made to include the case in the modelling. For example, when body weight was missing we used a default body weight of 60 kg for adults. For children, we used the default values from WHO 2006 and 2007 [15], two years: 11 kg, four years: 17 kg, seven years: 22.8 kg, eight years: 25.4 kg, 7–14 years: 31.2 kg.

For none of the outbreaks was the toxin profile available, as the mouse bioassay was the only method used for quantify cation of contaminated shellfish except one study [8] based on HPLC-FLD analysis (see Table A1 in the Appendix). In 3 other studies, some samples of contaminated shellfish were analysed by HPLC-FLD, but only for identification of PSTs analogues [11,12,13]. In order to convert exposure estimates from mouse unit (MU) to µg STX eq./kg b.w., we used a conversion factor of 0.18 [2,9,10].

At the end of the selection step, data eligible for modelling were collected in a database and included 191 exposed individuals from 16 studies [8,9,10,11,12,13,16,17,18,19,20,21,22,23,24,25], as presented in Table 5:

- 149 with symptoms; and
- 42 without symptoms.

### 4.2. Symptoms Classification

- **An ordinal scale** was used from 1 to 4 based on EFSA 2009 [2] (citing (Prakash et al., 1971) [22]) to classify the symptoms of PSP:

1. **mild symptoms**: tingling sensation or numbness around the lips gradually spreading to the face and neck, a prickly sensation in fingertips and toes, headache, dizziness, and nausea.
2. **moderate symptoms**: incoherent speech, general weakness, slight respiratory difficulty, and rapid pulse.
3. **severe symptoms**: muscular paralysis, pronounced respiratory difficulty.
4. **fatal cases**: death is caused by respiratory paralysis in the absence of artificial respiration.

Each individual was given the category associated to the most severe symptom, or 0 in the case of no symptoms. Details of symptoms by category are given in Table 6.

### 4.3. Dose-Response Modelling

#### 4.3.1. Selection of the Data for the Dose-Response Relationship

We explored the relationship between dose and response (as quantitative variable) graphically and by the estimate of *R*^2^ (coefficient of determination).

As a first step, one study was excluded ((McCollum et al., 1968) [20]) for the dose-response relationship due to aberrant values (exposure for individuals without symptoms were higher than exposure for individuals with symptoms).

As a second step, all the data from the remaining 15 selected studies were plotted as the category of symptoms (0: no symptoms; 1: mild symptoms; 2: moderate symptoms; 3: severe symptoms; 4: death) in function of the dose of PSTs ingested (in µg STX eq./kg b.w.) expressed in Log10. However, graphically no clear dose-response relationship was found.

As a third step, the 15 selected studies were classified according to the level of confidence in the reporting into three classes: low/medium/high (Table 7). The criteria for studies of high level of confidence were: very few assumptions to estimate the dose, analysis in leftover shellfish, and declared amount consumed. For studies of low level of confidence, the criteria were: many assumptions to estimate the dose, analysis of other shellfish than consumed, and the amount consumed not clear. In-between studies were classified as medium. When only studies of a high level of confidence were considered, the total number of data points was too low to establish a dose-response relationship. When including studies of high and medium level of confidence, the results were no better than with all the studies (high, medium, and low level of confidence).

**Figure 6:**
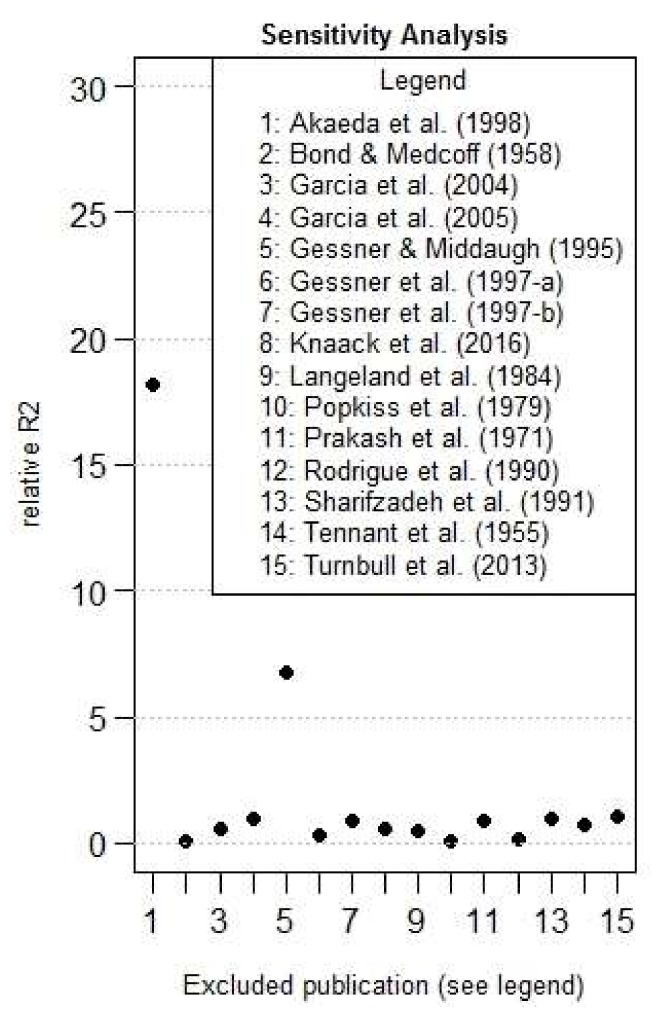
Relative *R*^2^ in function of the excluded publication (*n* = 15).

As a fourth step, we made a rough sensitivity analysis based on the estimate of *R*^2^, making the estimate of the *R*^2^ with and without each studies. The relative influence is determined by the ratio of the *R*^2^ without a specific study, with the *R*^2^ of the complete dataset, and is shown in Figure 6. Two studies were determined to be particularly influential in comparison with others: Akaeda et al., 1998 [9] and Gessner and Middaugh, 1995 [18], studies numbered 1 and 5 in Figure 6.

At the end, the *R*^2^ value with the complete dataset was estimated at 0.0074, increased to 0.13 without the Akaeda study [9], and to 0.299 without studies [9,18].

**Figure 7:**
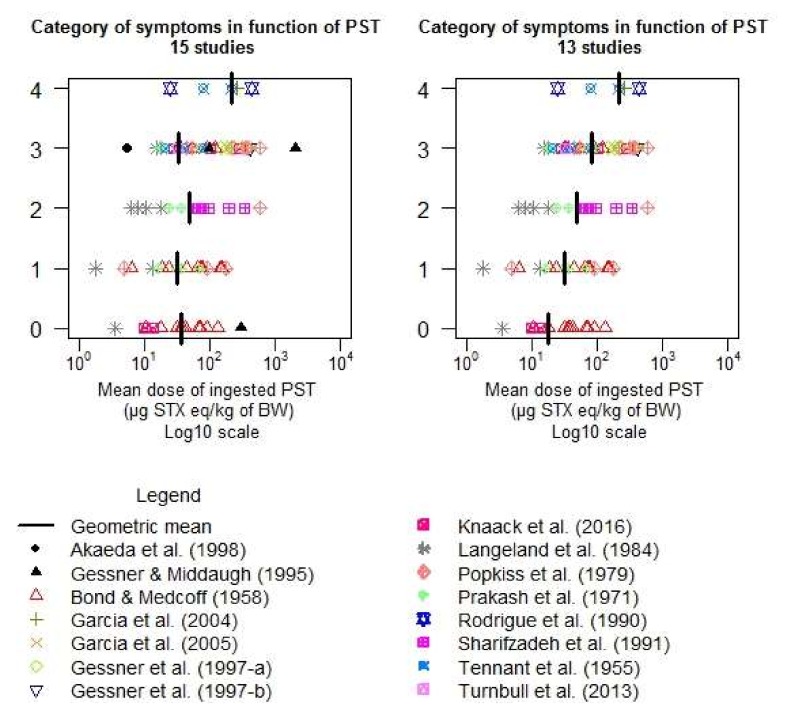
Category of symptoms in the key studies in function of exposure (*n* = 15, left, without Akaeda et al. 1998 [9]; Gessner and Middaugh 1995 [18] *n* = 13).

Those two influential studies, whose data are particularly far from the mean of their category, were removed from the analysis (Figure 7). With the new dataset of 13 studies, a significant linear dose-response relationship was found (*p*-value < 0.001).

#### 4.3.2. Descriptive Analysis of the Data for the Dose-Response Relationship

The data for the modelling included 143 exposed individuals: 113 with symptoms and 30 without symptoms (three doses from the study of Bond and Medcoff (1958) [16] could not be estimated).

**Figure 8:**
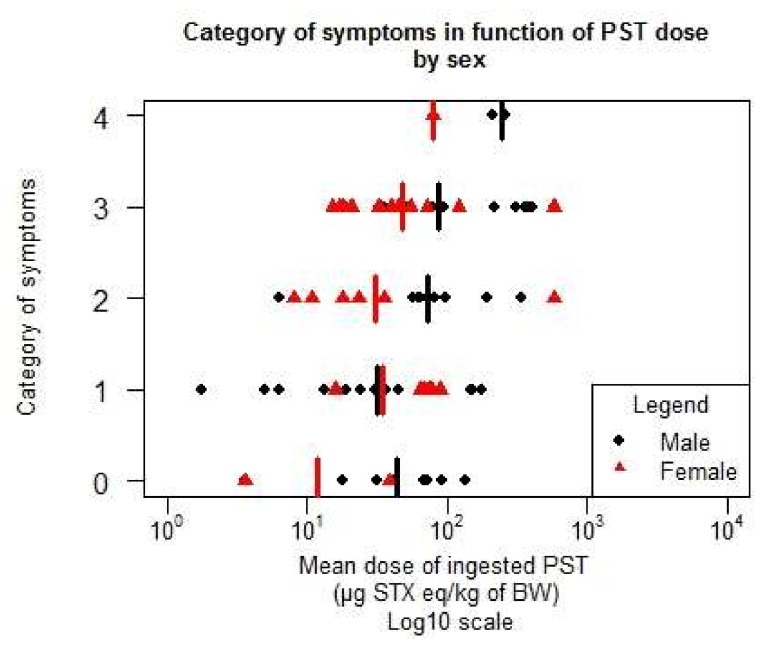
Distribution of the symptoms by sex in terms of the function of the mean ingested dose (number of males = 70, number of females = 41).

**Figure 9:**
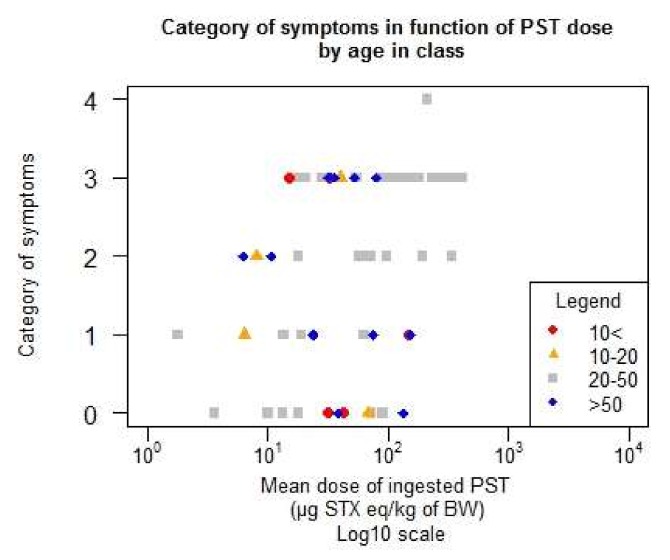
Distribution of the symptoms by age in class in terms of the function of the mean ingested dose (number of individuals < 10 years old = 5, number of individuals 10–20 = 6, number of individuals 20–50 = 36, number of individuals > 50 = 14).

The detailed data are given in Table A2 in the Appendix. Figure 8 gives the distribution of the data according to the sex and Figure 9 to the age of the individuals. Females seem to have more symptoms than men at a lower level of dose. No particular trend with age can be deduced from Figure 9.

#### 4.3.3. Ordinal Modelling of the Dose-Response Relationship

Level of severity of symptoms is classified as ordinal data from 0 to 4.

To analyse this ordinal data, we used the family of cumulative link models (CLM) [26,27,28]. This kind of modelling is often used to test medical treatments [29,30] and body condition scores linked to environmental factors [31,32,33]. The response variable in the model is the ordered category of symptoms.

The general equation of the cumulative link model is:

(1) $$F\left(P(Y\leq k\;|X\right))=a_{0,k}+a_{1,k}X_{1}+\ldots +a_{J,k}X_{J}$$

With *F* being the link function;

*X* is the set of *J* explanatory variables (*X*_1_, *X*_2_, …, *X_J_*);

*Y* is the response variable;

*k*_1_ < *k*_2_ < … < *k_K_* is the different levels of the variable *k* (ordinal scale of severity of symptoms);

P(Y≤k |X) is the probability of *Y* to reach a lower or equal level than *k* knowing *X*;

*a_i_*_,*k*_ is the slope associated to the variable *i* for the level *k* of the ordinal variable; and

*a*_0,*k*_ is the intercept associated with the level *k* of the ordinal variable.

The hypothesis of proportional-odds assumption, also called parallel-slopes assumption, was tested for the logistic and probit models by comparison with the multinomial model and cannot be rejected. The Figure 7 also shows that, graphically, only for the last stage, this hypothesis can be further discussed.

The effect of explanative variables is then assumed not to depend on a considered level. The equation can be simplified, such as in the Equation (2):

(2) $$F\left(P(Y\leq k\;|X\right))=a_{0,k}+a_{1}X_{1}+\ldots +a_{J}X_{J}$$

*a_i_*_,_ is the slope associated to the variable *i*.

The different models with different link function (logistic/probit) were compared using Akaike information criteria, the best model being the one with the lowest AIC. However, according to Burnham and Anderson (2004) [34], the difference of AICs below two units may be regarded as describing the data equally well. To show a statistical evidence of dose-effect, AIC should be lower than AIC of the null model minus 2 (AIC_nul_-2) (34). The thresholds (intercepts) of all ordinal models were chosen to be flexible.

The effects of explanatory variables, such as dose, sex, and age (centred and reduced), were tested as fixed effects. The publication effect was tested as a random effect, in an ordinal cumulative link mixed model (CLMM). The effects of explanatory variables of nested models was selected with a log likelihood ratio test. The selection of the non–nested model was tested by the Akaike information criteria and by the overall model fit, in particular at low doses. The model fit was checked by a comparison between the observed and predicted results. Statistical analyses were done with the R 3.4.3 (R development Core Team) *ordinal* package (last release 28 June 2015, Christensen 2013). From the results of the best–fitted model, prediction curves and their 95% confidence interval can be established.

Different critical doses were estimated for different levels of probability of symptoms, such as 10%, 5%, and 1%. Reference values, for health effects (other than cancer) were initially based on the no-observed-adverse level (NOAEL) or on the lowest-adverse-effect level (LOAEL). However, severe limitations of the NOAEL approach were identified by US-EPA [35] and EFSA [36,37]. The benchmark dose (BMD) approach was initially proposed by Crump (1984) [38]. BMD is the dose corresponding to a specified increase in the extra response of an exposed group in comparison with a control group. Even if already studied [39], there is no official approach to establish BMD from ordinal data.

The background level is established from the fitted dose-response curve, but is expected to be close to zero, knowing the specificity of symptoms linked to PSTs. Thus, from different risks (probability) of each kind of symptom, we can back-calculate the corresponding dose. Even more, we can calculate from the fitting the probability of no-symptoms, more than level 1 symptoms, more than level 2 symptoms, more than level 3 symptoms, and in final death risk.

The lower critical doses (LCD) was estimated with the lower-bound of the 95% confidence critical dose, and is comparable to the BMDL approach (lower bound of the BMD confidence interval). The upper critical dose (UCD close to the concept of the BMDU) is the upper-bound of the 95% confidence critical dose. The ratio UCD/LCD (close to the concept of BMDU/BMDL) describes the uncertainty of the critical dose estimate [36].

## Figures and Tables

**Table 1:** AIC (Akaike information criterion) and the *p*-value likelihood ratio test for the logit and probit models.

Logit			Probit		
Explanatory Variable	*p*-Value	AIC	Explanatory Variable	*p*-Value	AIC
Null model	-	439.1776	Null model	-	439.1776
Dose	10^−10^	402.4831	Dose	10^−10^	402.0386
Standardized Log10(Dose)	10^−12^	390.8066	Standardized Log10(Dose)	10^−13^	389.3283

**Table 2:** Parameters of the final model (cumulative link model) (dose is log10 centre reduced: mean = 1.6687, sd = 0.5395).

Parameter	Estimate	Standard Error	Z Value
Dose	0.713	0.1023	6.976
Threshold 0/1	−0.9976	0.1319	−7.562
Threshold 1/2	−0.1731	0.1132	−1.528
Threshold 2/3	0.3500	0.1151	3.04
Threshold 3/4	1.9031	0.2000	9.514

**Table 3:** Probability of showing symptoms (or not) as a function of the dose of ingested PSTs (log10 scale) with five categories of symptoms.

Dose of PSTs (µg STX eq/kg b.w.)	Prob. No Symptoms	Prob. Mild Symptoms	Prob. Moderate Symptoms	Prob. Severe Symptoms	Prob. Death	
1	88.673%	9.2229%	1.57%	0.526%	0.002%	100%
10	45.49%	30.66%	12.99%	10.59%	0.27%	100%
100	7.55%	19.5%	19.43%	46.37%	7.15%	100%
1000	0.3%	2.4%	5.3%	47.7%	44.3%	100%
10,000	0.002%	0.054%	0.257%	11.585%	88.102%	100%

**Table 4:** Critical doses (CD), lower critical doses (LCD), and upper critical doses (UCD) estimated for each category of symptoms and for different levels of risk (LR). All doses are expressed in µg STX eq/kg b.w.

	**Category of Symptoms > 0**			**Category of Symptoms > 1**		
LR	LCD	CD	UCD	LCD	CD	UCD
10%	**0.37**	0.88	2.58	**1.85**	3.71	7.855
5%	0.195	0.47	1.84	0.94	1.97	5.25
1%	0.06	0.14	1.24	0.275	0.6	3.095
	**Category of Symptoms > 2**			**Category of Symptoms > 3**		
LR	LCD	CD	UCD	LCD	CD	UCD
10%	**5.16**	9.21	16.72	**82.2**	137.63	341.86
5%	2.58	4.89	10.68	43.56	73.11	180.62
1%	0.74	1.49	5.69	12.69	24.74	68.92

Category of symptoms: 0 = no symptoms; 1 = mild symptoms; 2 = moderate symptoms; 3 = severe symptoms; 4 = death.

**Table 5:** Description of the studies at the end of the selection process according to the number of exposed individuals and the number of individuals with or without symptoms.

-	Number of Exposed Individuals	Number of Individuals with Symptoms	Number of Individuals without Symptoms	Country or State	Shellfish	Data on Body Weight	Estimation of Dose of PSTs Ingested	Reference
Akaeda et al. (1998)	26	26	0	Japan	Oysters ^1^	Default	Our study	[9]
Bond and Medcoff (1958)	46	20	26	Canada	Clams ^2^	Default	Authors (*by pers*.) ^14^	[16]
Garcia et al. (2004)	2	2	0	Chile	Mussels ^3^	Default	Our study	[17]
Garcia et al. (2005)	4	4	0	Chile	Mussels ^3^	Declared ^13^	Authors and our study	[8]
Gessner and Middaugh (1995)	12	2	10	Alaska	Clams, mussels, cockles ^4^	Default	Authors (*by pers*.)	[18]
Gessner et al. (1997-a)	6	6	0	Alaska	Mussels ^5^	Declared	Authors	[11]
Gessner et al. (1997-b)	1	1	0	Alaska	Mussels ^6^	Declared	Authors	[12]
Knaack et al. (2016)	5	3	2	Alaska	Mussels or cockles ^6^	Default	Our study	[19]
Langeland et al. (1984)	10	8	2	Norway	Mussels ^6^	Declared	Authors	[10]
McCollum et al. (1968)	7	5	2	UK	Mussels ^6^	Default	Authors (*by pers*.)	[20]
Popkiss et al. (1979)	16	16	0	South Africa	Mussels ^7^	Default	Authors (*by pers*.)	[21]
Prakash et al. (1971)	37	37	0	Canada	Clams, mussels or whelks ^8^	Default	Authors ^15^	[22]
Rodrigue et al. (1990)	5	5	0	Guatemala	Clams ^9^	Declared	Authors	[13]
Sharifzadeh et al. (1991)	6	6	0	Massachusetts	Mussels ^10^	Default	Our study	[23]
Tennant et al. (1955)	7	7	0	Canada	Clams ^11^	Default	Our study	[24]
Turnbull et al. (2013)	1	1	0	Australia	Mussels ^12^	Default	Authors (*by pers*.)	[25]

^1^ Oysters *Crassostrea gigas*; ^2^ Bar clams (*Spisula solidissima*) or soft-shell clams (*Mya arenaria*); ^3^ Ribbed mussels (*Aulacomya ater*);^4^ Butter clams (*Saxidomus giganteus*), mussels (*Mytilus edulis or californiacus*), cockles (*Clinocardium nuttalli*), razor clams (*Siliqua patula*), littleneck clams (*Protothaca staminea*) or other unidentifed species; ^5^ Mussels (*Mytilus edulis or californiacus*); ^6^ species not indicated;); ^7^ Black mussels (*Choromytilus meridionamis*); ^8^ soft-shellclams, rough whelks, species not indicated; ^9^ clams (*Amphichaena kindermani*); ^10^ Mussels (*Mytilus edulis*); ^11^ Soft-shell clams (*Mya arenaria*); ^12^ Mussels (*Mytilus galloprovincialis*); ^13^ mean body weight of four men; ^14^ ingested dose of PSTs recalculated in our study because the authors assumed that only 30% of the amount of toxins remained after cooking; ^15^ average and range by person, by category of symptoms.

**Table 6:** Classification of the symptoms.

Category of Symptoms	Symptoms
1	Headache
1	Paresthesia (abnormal sensation such as tingling, pricking, numbness)
1	Dizziness (impairment in spatial perception and stability)
1	Nausea, vomiting
1	Vertigo
2	Incoherent speech
2	Nystagmus (involuntary eye movement)
2	Rapid pulse
2	Ataxia (lack of voluntary coordination of muscle movements)
2	Dyspnea (shortness of breath)
2	Backache
3	Dysarthria (motor speech disorder)
3	Dysphagia (difficulty in swallowing)
3	Apnea (suspension of breathing)
3	Weakness of arms and legs
3	Pronounced respiratory difficulties
3	Muscular paralysis
3	Respiratory arrest (without death)
4	Death

**Table 7:** Classification of the 15 selected studies according to the level of confidence in the reporting.

Level of Confidence	Low	Medium	High
Studies	Gessner et al. (1997-b)	Akaeda et al. (1998)	Gessner et al. (1997-a)
	Prakash et al. (1971)	Bond and Medcoff (1958)	Knaack et al. (2016)
	Rodrigue et al. (1990)	Garcia et al. (2004)	-
	Sharifzadeh et al. (1991)	Garcia et al. (2005)	-
	Tennant et al. (1955)	Gessner and Middaugh (1995)	-
	Turnbull et al. (2013)	Langeland et al. (1984)	-
	-	Popkiss et al. (1979)	-

## Appendix A

**Table A1:** Additional information of selected studies regarding the dinoflagellate species involved in the outbreak, and the analytic method used for PSTs quantification.

Publication	Country or State	Dinoflagellate Species as Reported in the Publication	Toxin Quantification/Profile Provided
Bond and Medcoff (1958)	Canada	Suspected *Gonyaulax tamarensis*	Mouse bioassay in shellfish
Garcia et al. (2004)	Chile	Suspected *Alexandrium catenella*	Mouse bioassay in shellfish HPLC-FLD in human tissues
Garcia et al. (2005)	Chile	*Alexandrium catenella*	HPCL-FLD in shellfish (quantification, sum of GTX2 and 3)
Gessner et al. (1997-a)	Alaska	Not reported	Mouse bioassay for all samples + HPLC-FLD in some cooked mussels concerning 2 cases for analogues identification HPLC-FLD in humans tissues
Gessner et al. (1997-b)	Alaska	Not reported	Mouse bioassay in shellfish identification of the most prevalent analogues by HPLC-FLD
Knaack et al. (2016)	Alaska	Not reported	Mouse bioassay in shellfish HPLCMS/MS in human samples
Langeland et al. (1984)	Norway	*Gonyaulax excavata*	Mouse bioassay in shellfish
Popkiss et al. (1979)	South Africa	*Gonyaulax catenella*	Mouse bioassay in shellfish
Prakash et al. (1971)	Canada	Suspected *Gonyaulax tamarensis*	Mouse bioassay in shellfish
Rodrigue et al. (1990)	Guatemala	*Pyridinium bahamense*	Mouse bioassay in shellfish samples Identification of the most prevalent analogues by HPLC-FLD in one sample (STX B1, STX, neoSTX)
Sharifzadeh et al. (1991)	Massachusetts	Not reported	Mouse bioassay in shellfish
Tennant et al. (1955)	Canada	Suspected *Gonyaulax tamarensis*	Mouse bioassay in shellfish
Turnbull et al. (2013)	Australia	*Gymnodinium catenetum*	Mouse bioassay in shellfish

**Table A2:** Detailed database for modelling: NA = not available; estim_weight: default body weight when necessary, dose in µg STX eq/kg b.w., Category of symptoms: 0 (no symptoms), 1 (mild symptoms), 2 (moderate symptoms), 3 (severe symptoms), 4 (death).

Individual	Publication	Sex	Age	Weight	Estim_Weight	Dose	Category of Symptoms
1	Bond & Medcoff (1958)	F	35	NA	60	55.0	3
2	Bond & Medcoff (1958)	F	30	NA	60	122.0	3
3	Bond & Medcoff (1958)	M	45	NA	60	91.0	3
4	Bond & Medcoff (1958)	F	2	NA	11	32.7	3
5	Bond & Medcoff (1958)	M	63	NA	60	36.0	3
6	Bond & Medcoff (1958)	F	50	NA	60	64.0	1
7	Bond & Medcoff (1958)	M	65	NA	60	155.1	1
8	Bond & Medcoff (1958)	M	30	NA	60	32.4	3
9	Bond & Medcoff (1958)	F	NA	NA	60	77.4	1
10	Bond & Medcoff (1958)	M	20	NA	60	6.5	1
11	Bond & Medcoff (1958)	M	NA	NA	60	77.4	1
12	Bond & Medcoff (1958)	F	55	NA	60	75.6	1
13	Bond & Medcoff (1958)	M	50	NA	60	19.0	1
14	Bond & Medcoff (1958)	M	60	NA	60	24.0	1
15	Bond & Medcoff (1958)	M	8	NA	25.4	148.8	1
16	Bond & Medcoff (1958)	M	NA	NA	60	73.0	1
17	Bond & Medcoff (1958)	M	NA	NA	60	45.0	1
18	Bond & Medcoff (1958)	M	50	NA	60	18.0	0
19	Bond & Medcoff (1958)	M	10.5	NA	31.2	69.2	0
20	Bond & Medcoff (1958)	M	10.5	NA	31.2	69.2	0
21	Bond & Medcoff (1958)	M	10.5	NA	31.2	69.2	0
22	Bond & Medcoff (1958)	M	30	NA	60	73.0	0
23	Bond & Medcoff (1958)	M	4	NA	17	42.4	0
24	Bond & Medcoff (1958)	M	7	NA	22.8	31.6	0
25	Bond & Medcoff (1958)	M	40	NA	60	91.0	0
26	Bond & Medcoff (1958)	M	55	NA	60	134.0	0
27	Bond & Medcoff (1958)	NA	NA	NA	60	36.0	0
28	Bond & Medcoff (1958)	NA	NA	NA	60	10.8	0
29	Bond & Medcoff (1958)	NA	NA	NA	60	10.8	0
30	Bond & Medcoff (1958)	NA	NA	NA	60	10.8	0
31	Bond & Medcoff (1958)	NA	NA	NA	60	10.8	0
32	Bond & Medcoff (1958)	NA	NA	NA	60	10.8	0
33	Bond & Medcoff (1958)	NA	NA	NA	60	10.8	0
34	Bond & Medcoff (1958)	NA	NA	NA	60	10.8	0
35	Bond & Medcoff (1958)	NA	NA	NA	60	10.8	0
36	Bond & Medcoff (1958)	NA	NA	NA	60	10.8	0
37	Bond & Medcoff (1958)	NA	NA	NA	60	10.8	0
38	Bond & Medcoff (1958)	NA	NA	NA	60	10.8	0
39	Bond & Medcoff (1958)	NA	NA	NA	60	10.8	0
40	Bond & Medcoff (1958)	NA	NA	NA	60	10.8	0
41	Bond & Medcoff (1958)	NA	NA	NA	60	10.8	0
42	Bond & Medcoff (1958)	NA	NA	NA	60	10.8	0
43	Bond & Medcoff (1958)	F	60	NA	60	38.7	0
44	Garcia et al. (2004)	M	NA	NA	60	263.0	4
45	Garcia et al. (2004)	M	NA	NA	60	263.0	4
46	Garcia et al. (2005)	M	60	70.2	NA	52.9	3
47	Garcia et al. (2005)	M	62	70.2	NA	52.9	3
48	Garcia et al. (2005)	M	64	70.2	NA	52.9	3
49	Garcia et al. (2005)	M	66	70.2	NA	52.9	3
50	Gessner et al. (1997-a)	NA	37	NA	55	411.0	3
51	Gessner et al. (1997-a)	NA	37	NA	55	340.0	3
52	Gessner et al. (1997-a)	NA	37	NA	55	240.0	3
53	Gessner et al. (1997-a)	NA	37	NA	55	230.0	3
54	Gessner et al. (1997-a)	NA	37	NA	55	184.0	3
55	Gessner et al. (1997-a)	NA	37	NA	55	150.0	3
56	Gessner et al. (1997-b)	M	28	91	NA	411.0	3
57	Knaack et al. (2016)	NA	41.5	NA	60	285.4	3
58	Knaack et al. (2016)	NA	41.5	NA	60	13.4	0
59	Knaack et al. (2016)	NA	41.5	NA	60	10.1	0
60	Knaack et al. (2016)	NA	41.5	NA	60	100.7	3
61	Knaack et al. (2016)	NA	41.5	NA	60	28.6	3
62	Langeland et al. (1984)	M	26	80	NA	18.0	3
63	Langeland et al. (1984)	F	26	55	NA	18.0	2
64	Langeland et al. (1984)	F	6	28	NA	15.3	3
65	Langeland et al. (1984)	M	29	73	NA	13.5	1
66	Langeland et al. (1984)	F	57	51	NA	10.8	2
67	Langeland et al. (1984)	F	16	52	NA	8.1	2
68	Langeland et al. (1984)	M	54	85	NA	6.3	2
69	Langeland et al. (1984)	F	37	73	NA	3.6	0
70	Langeland et al. (1984)	M	37	70	NA	3.6	0
71	Langeland et al. (1984)	M	36	85	NA	1.8	1
72	Popkiss et al. (1979)	M	NA	NA	60	219.0	3
73	Popkiss et al. (1979)	M	NA	NA	60	383.0	3
74	Popkiss et al. (1979)	M	NA	NA	60	383.0	3
75	Popkiss et al. (1979)	M	NA	NA	60	55.0	3
76	Popkiss et al. (1979)	M	NA	NA	60	360.0	3
77	Popkiss et al. (1979)	M	NA	NA	60	54.0	3
78	Popkiss et al. (1979)	M	NA	NA	60	315.0	3
79	Popkiss et al. (1979)	F	NA	NA	60	45.0	3
80	Popkiss et al. (1979)	M	NA	NA	60	585.0	3
81	Popkiss et al. (1979)	F	NA	NA	60	585.0	2
82	Popkiss et al. (1979)	F	NA	NA	60	585.0	3
83	Popkiss et al. (1979)	F	NA	NA	60	585.0	3
84	Popkiss et al. (1979)	M	NA	NA	60	180.0	1
85	Popkiss et al. (1979)	F	NA	NA	60	90.0	1
86	Popkiss et al. (1979)	F	NA	NA	60	18.0	3
87	Popkiss et al. (1979)	M	NA	NA	60	5.0	1
88	Prakash et al. (1971)	M	NA	NA	60	30.7	1
89	Prakash et al. (1971)	M	NA	NA	60	30.7	1
90	Prakash et al. (1971)	M	NA	NA	60	30.7	1
91	Prakash et al. (1971)	M	NA	NA	60	30.7	1
92	Prakash et al. (1971)	F	NA	NA	60	16.0	1
93	Prakash et al. (1971)	F	NA	NA	60	16.0	1
94	Prakash et al. (1971)	F	NA	NA	60	16.0	1
95	Prakash et al. (1971)	F	NA	NA	60	16.0	1
96	Prakash et al. (1971)	F	NA	NA	60	16.0	1
97	Prakash et al. (1971)	F	NA	NA	60	16.0	1
98	Prakash et al. (1971)	M	NA	NA	60	36.9	1
99	Prakash et al. (1971)	M	NA	NA	60	36.9	1
100	Prakash et al. (1971)	M	NA	NA	60	36.9	1
101	Prakash et al. (1971)	M	NA	NA	60	36.9	1
102	Prakash et al. (1971)	M	NA	NA	60	36.9	1
103	Prakash et al. (1971)	M	NA	NA	60	36.9	1
104	Prakash et al. (1971)	F	NA	NA	60	68.0	1
105	Prakash et al. (1971)	F	NA	NA	60	68.0	1
106	Prakash et al. (1971)	M	NA	NA	60	62.7	2
107	Prakash et al. (1971)	M	NA	NA	60	62.7	2
108	Prakash et al. (1971)	M	NA	NA	60	62.7	2
109	Prakash et al. (1971)	F	NA	NA	60	36.0	2
110	Prakash et al. (1971)	F	NA	NA	60	36.0	2
111	Prakash et al. (1971)	F	NA	NA	60	36.0	2
112	Prakash et al. (1971)	F	NA	NA	60	36.0	2
113	Prakash et al. (1971)	M	NA	NA	60	81.1	2
114	Prakash et al. (1971)	M	NA	NA	60	81.1	2
115	Prakash et al. (1971)	M	NA	NA	60	81.1	2
116	Prakash et al. (1971)	F	NA	NA	60	23.6	2
117	Prakash et al. (1971)	F	NA	NA	60	23.6	2
118	Prakash et al. (1971)	F	NA	NA	60	23.6	2
119	Prakash et al. (1971)	M	NA	NA	60	96.0	3
120	Prakash et al. (1971)	F	NA	NA	60	72.0	3
121	Prakash et al. (1971)	M	NA	NA	60	19.2	3
122	Prakash et al. (1971)	M	NA	NA	60	19.2	3
123	Prakash et al. (1971)	F	NA	NA	60	17.2	3
124	Prakash et al. (1971)	F	NA	NA	60	17.2	3
125	Rodrigue et al. (1990)	NA	NA	25	NA	25.2	4
126	Rodrigue et al. (1990)	NA	NA	NA	60	437.0	4
127	Rodrigue et al. (1990)	NA	NA	NA	60	437.0	4
128	Rodrigue et al. (1990)	NA	NA	NA	60	437.0	4
129	Rodrigue et al. (1990)	NA	NA	NA	60	437.0	4
130	Sharifzadeh et al. (1991)	M	35.5	NA	60	65.1	2
131	Sharifzadeh et al. (1991)	M	35.5	NA	60	57.0	2
132	Sharifzadeh et al. (1991)	M	35.5	NA	60	73.2	2
133	Sharifzadeh et al. (1991)	M	35.5	NA	60	97.6	2
134	Sharifzadeh et al. (1991)	M	35.5	NA	60	195.2	2
135	Sharifzadeh et al. (1991)	M	35.5	NA	60	341.6	2
136	Tennant et al. (1955)	M	36	NA	60	212.6	4
137	Tennant et al. (1955)	F	NA	NA	60	79.8	4
138	Tennant et al. (1955)	M	69	NA	60	79.8	3
139	Tennant et al. (1955)	F	60	NA	60	32.3	3
140	Tennant et al. (1955)	F	34	NA	60	32.3	3
141	Tennant et al. (1955)	F	27	NA	60	20.9	3
142	Tennant et al. (1955)	F	12	NA	31.2	40.2	3
143	Turnbull et al. (2013)	M	NA	NA	60	32.6	3
